# Effects of physiological stage and season on infrared thermograms of different body areas of dairy cows raised under tropical conditions

**DOI:** 10.21451/1984-3143-AR2017-0023

**Published:** 2019-10-23

**Authors:** Fernanda Luiza Guinossi Barbosa Deak, Marcelo George Mungai Chacur, Camila Dutra de Souza, Isamara Batata Andrade, Gabriela Figueredo Cornacini, Alexandre Rossetto Garcia, Luís Roberto Almeida Gabriel

**Affiliations:** 1 Laboratory of Animal Reproduction, Faculty of Agrarian Sciences, University of West Paulista (UNOESTE), Presidente Prudente, SP, Brazil.; 2 EMBRAPA – Pecuária Sudeste, São Carlos, SP, Brazil.; 3 São Paulo State University (UNESP), School of Sciences and Engineering, Tupã, SP, Brazil.

**Keywords:** dairy cattle, IRT, reproduction, pregnancy

## Abstract

The objective of this study was to investigate the influence of season and pregnancy stage on the temperature of various body areas of Holstein cows using digital infrared thermography, an effective and non-invasive technique. The temperature was recorded at several areas of the body surface to determine the most reliable body area for measurement of rectal temperature in pregnant and non-pregnant animals. Holstein cows (n = 24) were divided into groups according to their physiological stage. The experimental period was 365 days, containing a dry (April-September) and rainy (October-March) season, with parameters measured every 28 days. Thermographic data for different body areas, rectal thermometry, ultrasonography, and climatic data were collected between 7:00 and 9:00. Thermogram-recorded temperatures significantly differed (P < 0.05) between seasons and reproductive phases. Moreover, significant differences were noted between the temperatures of the flank, lateral udder, and perineal areas across seasons (P < 0.05). The udder, perineal, and rectal temperatures differed according to the reproductive phase (P < 0.05). Significant correlations (P < 0.01) were observed between reproductive phases and rectal, ocular globe, snout, flank, and perineum temperature. The body areas examined by thermographic imaging presented different temperatures, exhibiting physiological variation. Season and physiological stage influenced the temperature of body areas of milk cows.

## Introduction

The body temperature of dairy cows is a clinical parameter that assists in the diagnosis of disease and evaluation of physiological state, and body temperature measurements are used to identify animals in hyperthermy or with fevers (Poulse and McGuirk, 2009; Luzi *et al*., 2013; Barros *et al*., 2016; Chacur, 2017). Invasive techniques that capture and record body temperature in cattle are known; these include intra-ruminal devices (Rose-Dye *et al*., 2011) and subcutaneously implanted transmitters (Georg *et al*., 2009). The type of thermometer and thermometry technique used influences the variation noted in the measured rectal temperature (Suthar *et al*., 2011; Naylor *et al*., 2012).

Digital infrared thermography is a highly accurate and non-invasive imaging technique that detects infrared radiation emitted by the surface of an object or living being, forming a thermographic image or thermogram (Turner, 1991; Eddy *et al*., 2001; Ruediger *et al*., 2016). Thermograms represent the temperature of an object’s surface with color, with the hottest areas recorded as white or red and the coldest, as blue or black (Colak *et al*., 2008). Infrared thermography assists in the early diagnosis of lesions that cause pain and inflammation and thermograpy can be installed in milking parlors to monitor surface temperatures of different areas of the body of dairy cows (Alsaaod *et al*., 2015). It can also be coupled with water troughs to identify animals and record the temperature of the ocular region, which is highly correlated with rectal temperature (Stewart *et al*., 2005).

Digital infrared thermography is used as a complementary examination in the early diagnosis of subclinical and clinical mastitis (Colak *et al*., 2008; Poikalainen *et al*., 2012; Berry *et al*., 2003), the clinical evaluation of gestational phase (Bowers *et al*., 2009), and the detection of progesterone-related changes in body temperature in pregnant cows (Suthar *et al*., 2012).

The present study was performed because of the limited availability of data in the literature regarding dairy cows in the tropics regarding their surface body temperature in different seasons and stages of pregnancy. This study was based on the hypothesis that the temperature of body areas of Holstein cows is influenced by rainfall distribution (rainy season and dry season) and physiological stage.

The objective of this study was to investigate the influence of season and stage of pregnancy on the surface temperature of body areas of Holstein cows using digital infrared thermography, which is an effective and non-invasive technique. The purpose was to determine the most reliable surface area in relation to the rectal temperature of pregnant and non-pregnant animals.

## Materials and Methods

The experimental procedures were approved by the Committee on Ethics and Use of Animals in Experimentation (CEUA) of Universidade do Oeste Paulista-UNOESTE (University of West Paulista), under protocol 2918.

### Location of experiment and study animals

The experiment was carried out between March 2015 and March 2016 in the municipality of Ribeirão dos Índios (Brazil), 21º58′33″S, 51º51°39′05″W, 386 m asl. This region has a tropical climate, with hot and rainy summers, and winters with a lower rainfall index than that of the summer (1700 mm/year).

Lactation cows (*n* = 24) of the Holstein breed (pure by cross 31 × 32), predominantly having a white coat, aged 5.0 ± 1.3 years, were used in the following reproductive phases: 1 (1–95 days of pregnancy); 2 (96–190 days of pregnancy); 3 (191–285 days of pregnancy); 4 (immediate postpartum); and 5 (non-pregnant). The animals were separated according to their reproductive phase, with an average of four to five females per group, and each animal advanced to the next phase as gestation progressed. All animals were kept in a pasture of *Brachiaria decumbens* and received free access to corn silage, mineral mixture, and water. The experimental period was 365 days, during the dry season (March-September) and rainy season (October-March).

### Data collection and examinations

The cows were housed under a cement roof, and data were collected and tests performed in the following order: infrared digital thermography, ultrasonography mode B, climatic parameters at intervals of 28 days over 12 months.

### Infrared digital thermography

Images were captured with an infrared digital thermography (Flir E40®, Sweden) at a distance of 1 m from the animal, with images captured in the same order for all animals as follows: ocular globe and muzzle (George *et al*., 2014); pelvis; abdomen and thorax as described by Metzner *et al*. (2014); perineum (vulva, dorsal commissure of the vulva, one-third of the vulva, ventral commissure of the vulva, right and left ischium); and mammary gland (right and caudal lateral areas) as described by Talukder *et al*. (2014). Thermograms of each body area were carefully captured in clean areas and without direct sunlight, as described by Hoffman *et al*. (2013). The emitting focus of the thermographic camera was directed perpendicular to the anatomical regions examined. Thermograms were processed by the computer program Flir Tools 2.1®. 1, 2, and 3 present the thermograms of the body areas, representing the average temperature of each area.

**Figure 2:**
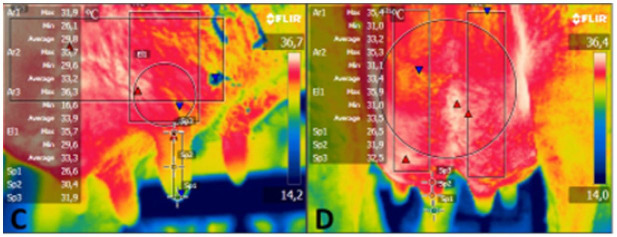
Thermograms with data of the temperatures obtained with the computer program in the areas examined: C. Right side of the anterior mammary room (demarcated area) and the anterior ceilings base, 1/3 middle and end (marked points); D. Caudal area of the mammary quarters (demarcated areas) and ceilings; base, 1/3 medium and end (marked points).

**Figure 3:**
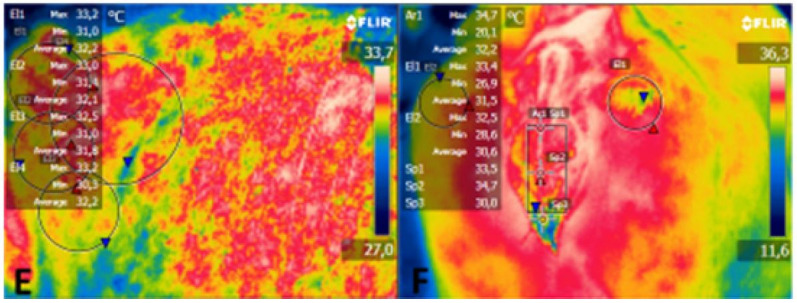
Thermograms with temperature data obtained from the computer program in the areas examined: E. Rigght lateral pelvis, 1/3 mid abdomen, 1/3 ventral absdomen and thorax (demarcated areas); F. Vulva (demarcated area), dorsal commissure of the vulva, 1/3 of the vulva, ventral commissure of the vulva (marked points), right and left ischium (demarcated area).

**Figure:**
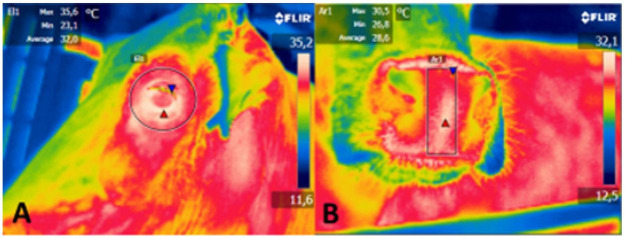
Thermograms with temperature data, obtained with the computer program in the areas examined: A. Ocular globe; B. Muzzle.

### Ultrasonography mode B

Transrectal ultrasound examinations (Aloka 500®, Japan) were performed with a 5 MHz linear transducer to diagnose pregnancy based on the day of artificial insemination and to confirm the continuity of pregnancy.

### Meteorological parameters

Meteorological parameters, including the wet bulb globe temperature, which is presented in degrees Celsius, black globe temperature (captures the emitted thermal radiation), ambient temperature, and relative humidity, were measured. These parameters were measured during each collection period every hour between 07:00 and 09:00 with a portable digital thermometer globe (Instrutemp HT30®, Brazil).

### Statistical analysis

In this study, an analysis of variance was conducted for the means of period of the year (dry and rainy) and reproductive phase: 1 (1-95 days of pregnancy); 2 (96–190 days of pregnancy); 3 (191-285 days of pregnancy); 4 (immediate postpartum); and 5 (non-pregnant). For temperatures of flank, lateral udder, perineum, and caudal udder, an analysis of variance was conducted with means of collected data (1 to 12). In addition, we analyzed the interactions between these factors for each variable. For the interaction, an analysis of variance was performed to compare the variables between each of the levels of a given factor. These were compared using Tukey’s test at a significance level of 5%. Correlations were measured for reproductive phase and rectal temperature, and body area temperatures and rectal temperature.

## Results

Table 1 presents the average values for each meteorological parameter (as gauged by three forms) and humidity for the rainy and dry seasons.

**Table 1:** Average values of meteorological parameters in each season.

Meteorological parameters	Season		
	Rainy	Dry	
WBGT (°C)	22.5 ± 1.9^a^	20.0 ± 4.4^b^
Ambient temperature (°C)	25.9 ± 3.1^a^	23.9 ± 5.1^b^
Black globe temperature (°C)	26.1 ± 3.4^a^	23.8 ± 5.5^b^
Relative humidity (%)	66.3 ± 14.0^a^	58.5 ± 14.5^b^
BGHI	74.53a	70.91^b^

Different letters in rows indicate significant differences (P < 0.05). WBGT: wet bulb globe temperature. BGHI: black globe humidity index.

The variable temperature of the flank, lateral udder, caudal udder, and perineum during collection times and averages and coefficients of variation are presented in Table 2. There was a significant difference (P < 0.05) between data collection periods, reproductive phases, and seasons. There were significant differences (P < 0.05) between data collection periods for different areas of the body: flank, lateral udder, caudal udder, and perineum on the thermograms (1, 2, and 3). Regarding the lateral udder, there was a significant difference (P < 0.05) between reproductive phases. For the temperature of the flank, lateral udder, and perineum, there was a significant difference (P < 0.05) between seasons (Tab. 2).

**Table 2:** Mean values for variables by collection time, reproductive phase, and season.

		Flank (ºC)	Lateral udder (ºC)	Perineum (ºC)	Caudal udder (ºC)
Collect	1	35.5 ± 1.4^a^	35.2 ± 1.6^ab^	35.2 ± 2.3^ab^	35.6 ± 1.4^ab^
	2	34.0 ± 0.7^def^	34.3 ± 1.4^cd^	34.4 ± 1.7^bc^	34.9 ± 1.2^cd^
	3	33.5 ± 1.5^fg^	34.1 ± 1.6^cd^	34.2 ± 3.0^bc^	34.9 ± 1.8^bcd^
	5	31.1 ± 1.9^h^	32.1 ± 2.5^e^	31.6 ± 4.5^e^	33.0 ± 1.8^f^
	6	34.4 ± 1.4^cd^	35.1 ± 1.6^ab^	33.8 ± 2.6^cd^	35.7 ± 1.4^a^
	7	33.1 ± 0.9^g^	33.9 ± 1.6^d^	32.8 ± 3.0^d^	34.1 ± 1.7^e^
	8	33.8 ± 0.8^ef^	34.2 ± 1.6^cd^	34.5 ± 2.2^abc^	34.8 ± 1.4^cd^
	9	35.1 ± 0.8^ab^	35.6 ± 1.3^a^	35.7 ± 1.7^a^	36.1 ± 1.3^a^
	10	34.6 ± 1.1^bc^	34.9 ± 1.5^b^	35.0 ± 2.0^ab^	35.5 ± 1.4^ab^
	11	33.6 ± 1.3^fg^	34.2 ± 1.5^cd^	34.1 ± 2.6^bc^	34.3 ± 3.2^de^
	12	34.3 ± 1.1^cde^	34.5 ± 1.7^bc^	34.6 ± 2.2^abc^	35.5 ± 1.4^abc^
Reproductive phase	1	33.9 ± 1.5^a^	34.3 ± 1.7^b^	34 ± 2.6^ab^	34.9 ± 1.7^ab^
	2	33.9 ± 1.8^a^	34.2 ± 2.1^b^	34.4 ± 2.7^a^	35.0 ± 1.8^ab^
	3	33.9 ± 1.6^a^	34.5 ± 1.6^ab^	34.6 ± 2.7^a^	34.8 ± 1.5^ab^
	4	34.1 ± 1.3^a^	34.9 ± 1.6^a^	34.2 ± 2.5^a^	35.1 ± 2.4 ^a^
	5	33.1 ± 1.9^b^	33.8 ± 1.8^c^	33.4 ± 3.9^b^	34.6 ± 1.7 ^b^
Season	Rainy	34.2 ± 1.2^a^	34.6 ± 1.6^a^	34.5 ± 2.4^a^	35.0 ± 1.8^a^
	Dry	33.3 ± 2.0^b^	33.9 ± 2.2^b^	33.5 ± 3.4^b^	34.8 ± 1.9^b^

Collections 1 to 12 had intervals of 28 days. Reproductive Phase: 1: 0–95 days of pregnancy; 2: 96–190 days of pregnancy; 3: 191–285 days of pregnancy; 4: immediately postpartum; 5: not pregnant. Different letters in columns indicate significant differences (P < 0.05).

The udder, perineum, and rectal temperatures differed according to the reproductive phase (P < 0.05) (Tab. 3).

**Table 3:** Mean values for rectal temperature collected by thermometry and udder and perineum temperatures collected with thermography during the different reproductive phases.

Reproductive phase	1	2	3	4	5
Udder	36.1 ± 1.2^ab^	36.6 ± 1.0^ab^	36.7 ± 0.9^ab^	36.1 ± 1.2^a^	35.8 ± 1.5^b^
Rectal	37.8 ± 0.4^b^	38.0 ± 0.5^ab^	38.0 ± 0.4^ab^	38.2 ± 0.4^a^	38.0 ± 0.5^ab^
Perineum	35.2 ± 1.2^ab^	35.3 ± 1.4^ab^	35.2 ± 1.2^ab^	35.8 ± 0.9^a^	34.6 ± 1.2^b^

Different letters in rows indicate significant differences (P < 0.05). Reproductive phase: 1: 0–95 days of pregnancy; 2: 96–190 days of pregnancy; 3: 191–285 days of pregnancy; 4: immediately postpartum; 5: not pregnant. Different letters in columns indicate significant differences (P < 0.05).

Table 4 compares the flank and udder temperatures during the dry and rainy seasons, revealing significant differences (P < 0.05) between seasons.

**Table 4:** Correlations between reproductive phase and rectal temperature, and body area temperatures and rectal temperature.

			Rectal temperature
		Reproductive phase	0.215*
		Ambient temperature	0.250*
		Relative humidity	-0.169*
		Lateral udder	0.357*
		Ocular globe (Eyeball)	0.316*
		Muzzle	0.234*
	
Flank		A1	0.352*
		A2	0.347*
		A3	0.331*
		A4	0.377*
		Average	0.357*
Perineum		Ischium	0.256*
		A1	0.324*
		SP1	0.213*
		SP2	0.218*
		SP3	0.273*
		Average	0.331*

* (P < 0.05) - Flank: A1: right side of the pelvis, A2: on-third mid-abdomen, A3: one-third ventral abdomen, and A4: thorax; perineum: A1: vulval temperature mean, SP1: dorsal commissure of the vulva, SP2: one-third of the vulva, SP3: ventral commissure of the vulva.

There were significant correlations (P < 0.01) between reproductive phases and rectal temperature, and rectal, eyeball, muzzle, flank, and perineum temperature (Tab. 4).

## Discussion

Regarding climatic factors, the results of the present study revealed that there were differences between seasons, corroborating the results reported by Berry *et al*. (2013) of positive correlations between temperatures measured with thermography and the ambient temperature and udder temperature.

In relation to black globe humidity index (BGHI), Baêta (1985) points out that values in the range of 74-78 characterize an alert state and values in the range of 79-84 characterize animals that are in danger, which may lead to low yield. In the present study, BGHI did not characterize a state of alertness.

Quantitative data for the ocular globe, muzzle, and flank temperatures were obtained as a measure of body temperature in pregnant Holstein cows, depending on the animal’s reproductive stage. The temperatures of several areas of the animal’s body surface were recorded to determine the most reliable area for measurement of rectal temperature. In the present study, the flank temperature was closest to that of the rectum.

In the present study, thermographic images were obtained as described by Alejandro *et al*. (2014), Metzner *et al*. (2014), Talukder *et al*. (2015), and Chacur *et al*. (2016), who used areas and points to measure the surface temperature of the skin. In addition, those authors performed the same noninvasive examination and reported it to be accurate. Ambient temperature and relative air humidity are measured at the time of thermographic examination to ensure that accurate data are collected (Montanholi *et al*., 2015).

The surface temperature of the body is influenced by the physiological health of the tissues and the oscillations caused by external factors (ambient temperature and relative humidity) (Gonçalves, 2013; Nikkhah, 2015). Light coats reflect heat, whereas dark coats absorb heat; therefore, capturing images of animals directly exposed to the sun should be avoided to obtain authentic thermographic images.

In the present study, the temperature was measured with a portable camera with cows in shadowed areas. Previously, Schaefer *et al*. (2012) explored the use of a thermal image using a camera attached to a water station, which was able to scan the animal’s eye. Thus, because the animals visited the water trough daily, it could help the farmer to identify animals with high temperatures. The identified animal could then be examined.

In the present study, there was a significant correlation between rectal temperature and ocular globe temperature. Validation of ocular globe temperature as an acceptable and accurate measure of rectal temperature has been conducted, and it is, therefore, a reliable measure of body temperature (Schaefer *et al*., 2012; Barros *et al*., 2016).

Temperatures collected for the flank, lateral udder, caudal udder, and perineal regions varied in relation to the periods in which they were collected. Such temperatures were similar to those described by Gil *et al*. (2013) and Chacur *et al*. (2016). With regard to the lateral udder and third of gestation variables, the temperature variations were 33.1 ± 3.9°C; similar results were obtained by Schaefer *et al*. (2012) and Okada *et al*. (2013) under different climatic conditions. There was a correlation between the temperature of the perineum and that of the muzzle, which is consistent with the findings of Metzner *et al*. (2014) and Talukder *et al*. (2015).

The gestation phase influenced temperature variation among animals, such that pregnant animals presented higher body temperatures in relation to non-pregnant cows. Suthar *et al*. (2012) and Rensis *et al*. (2015) reported that pregnant animals have a higher concentration of progesterone in their bloodstream compared with that of non-pregnant animals. This hormone has a thermogenic action and raises the body temperature of pregnant cows. The infrared images obtained in the present study were able to detect body temperature variations when comparing pregnant and non-pregnant cows. The variation in body temperature was 34.0 ± 2.4°C, which is similar to the values obtained by Gil *et al*. (2001) and Gabor (2016) who compared the body temperatures of pregnant and non-pregnant animals.

It can be concluded that different areas of the body examined by thermographic images presented different temperatures, showing physiological variation that may aid in the clinical evaluation of the areas examined. Body area surface temperatures of cows examined were positively correlated with rectal temperature. The most reliable surface temperature in relation to the rectal temperature was that of the flank when determined by infrared. Season and reproductive phase influenced the surface temperature of body areas.
